# Comparison of PSA value at last follow-up of patients who underwent low-dose rate brachytherapy and intensity-modulated radiation therapy for prostate cancer

**DOI:** 10.1186/s12885-017-3565-1

**Published:** 2017-08-25

**Authors:** Nobumichi Tanaka, Isao Asakawa, Yasushi Nakai, Makito Miyake, Satoshi Anai, Tomomi Fujii, Masatoshi Hasegawa, Noboru Konishi, Kiyohide Fujimoto

**Affiliations:** 1 Department of Urology, Nara Medical University, 840 Shijo-cho, Kashihara, Nara 634-8522 Japan; 2 Radiation Oncology, Nara Medical University, Kashihara, Nara Japan; 3 Pathology, Nara Medical University, Kashihara, Nara Japan

**Keywords:** Prostate cancer, Low-dose rate brachytherapy, IMRT, Biochemical recurrence-free rate, BED, Testosterone

## Abstract

**Background:**

To compare the PSA value at the last follow-up of patients who underwent prostate low-dose rate brachytherapy (LDR-BT) with that of patients who underwent intensity-modulated radiation therapy (IMRT).

**Methods:**

A total of 610 prostate cancer patients (cT1c-3bN0M0) were enrolled, and 445 of them underwent LDR-BT, while 165 received IMRT (74–76 Gy). The median follow-up period of these two groups was 75 months (LDR-BT) and 78 months (IMRT), respectively. We also evaluated the biochemical recurrence (BCR)-free rate using two definitions (Phoenix definition and PSA ≥ 0.2 ng/mL).

**Results:**

The percentage of patients who achieved PSA < 0.2 ng/mL at the last follow-up was 77.5% in the LDR-BT group and 49.7% in the IMRT group (*p* < 0.001). Among patients with a normal testosterone level at the last follow-up, the percentage of those who achieved PSA < 0.2 ng/mL at the last follow-up was 79.2% in the LDR-BT group and 32.1% in the IMRT group (*p* < 0.001). The 5-year BCR-free rate by the Phoenix definition in the IMRT and LDR-BT groups was 89.5 and 95.0% (*p* < 0.001), respectively. On the other hand, the 5-year BCR-free rate using the definition of PSA ≥ 0.2 ng/mL was 59.1 and 80.1% in the IMRT and LDR-BT groups, respectively (*p* < 0.001).

**Conclusions:**

The PSA value at the last follow-up of LDR-BT was significantly lower than that of IMRT, and this result was particularly marked in patients with a normal testosterone level at the last follow-up.

## Background

At present, the oncologic outcome of patients who undergo low-dose rate brachytherapy (LDR-BT) is similar to that of patients who undergo intensity-modulated radiation therapy (IMRT) or radical prostatectomy [1–6]. Generally, the Phoenix definition (nadir + 2 ng/mL) is used for patients who undergo definitive radiation therapy [7], while the cut-off value of prostate specific antigen (PSA) is 0.2 ng/mL for radical prostatectomy. Direct comparison of the biochemical recurrence rate between surgery and radiation therapy using these different definitions is questionable. The optimal PSA value after radiation therapy also leaves room for discussion. Critz et al. previously reported the long-term (median follow-up: 11 years) oncologic outcomes of LDR-BT in combination with external beam radiation therapy (EBRT) using the definition of PSA ≥ 0.2 ng/mL [8]. The disease-free survival rate was comparable to that of a radical prostatectomy series. They concluded that later recurrence is unlikely with PSA <0.2 ng/mL at 15 years after treatment. We have already reported the oncologic outcome of patients who underwent LDR-BT using the definition of PSA ≥ 0.2 ng/mL [9]. Approximately 80% of patients showed PSA < 0.2 ng/mL at the last follow-up. Unfortunately, the number of patients in our previous study was small (203 patients), and the influence of testosterone level was not taken into consideration. To elucidate the influence of testosterone level on PSA kinetics after radiation therapy, we conducted the present study evaluating the PSA value at the last follow-up in patients who had not only undergone LDR-BT, but also IMRT.

## Methods

A total of 1474 patients who were clinically diagnosed with prostate cancer (cT1c-3bN0M0) underwent definitive radiotherapy (LDR-BT:1074 patients, IMRT: 400 patients) in Nara Medical University Hospital between 2004 and 2016. Of these, a total of 610 patients (LDR-BT: 445 patients, IMRT: 165 patients) who underwent definitive radiotherapy between 2004 and 2011were enrolled. The patient characteristics are shown in Table 1. The median age, PSA value at diagnosis, and follow-up period in the LDR-BT group were 71 years (range: 48–83), 7.1 ng/mL (range: 3.1–43.6), and 75 months (range: 3–143), while those in the IMRT group were 74 years (range: 51–84), 14.1 ng/mL (range: 2.8–364), and 78 months (range: 18–125), respectively.

**Table 1:** Patient characteristics

	LDR-BT (*n* = 445)	IMRT (*n* = 165)	*p* value
Age (year)			
Median (range)	71 (48–83)	74 (51–84)	<0.001 ^b^
PSA at diagnosis (ng/mL)			
Median (range)	7.1 (3.1–43.6)	14.1 (2.8–364)	<0.001 ^c^
Biopsy Gleason score			
6 or less	245	37	
7	173	76	
8–10	27	52	<0.001 ^a^
Clinical T stage			
T1b/1c	0/238	2/44	
T2a/2b/2c	143/35/21	30/10/5	
T3a/3b	8/0	42/32	<0.001 ^a^
Neoadjuvant/Adjuvant ADT			
None	267	34	
neo-Ad (+)	141	24	
Ad (+)	10	14	
neo-Ad (+), Ad (+)	27	93	<0.001 ^a^
Risk stratification			
Low	184	17	
Intermediate	201	36	
High	60	112	<0.001 ^a^
BED (Gy2)			
Median (range)	199.2 (120.3–253.2)	148 (148–152)	<0.001 ^b^
Follow-up period			
Median (range)	75 (3–143)	78 (18–125)	0.118 ^b^
Prescribed dose (Gy)			
74		107	
76		58	
110	141		
145	97		
160	207		
EBRT			
No	300		
Yes	145		

*BED* biological effective dose, *ADT* androgen deprivation therapy, *Neoad* neoadjuvant, *Ad* adjuvant, *EBRT* external beam radiation therapy

^a^Chi-square test

^b^t-test

^c^Mann-Whitney U test

We compared the PSA value at the last follow-up between the LDR-BT and IMRT groups. To eliminate the influence of androgen deprivation therapy (ADT), we also compared the PSA value in both groups of patients with normal testosterone levels at the last follow-up. We defined a normal testosterone level as 1.75 ng/mL or higher, which is the standard level of our institution. We evaluated the PSA value at the last follow-up after at least 4 years, and also conducted univariate and multivariate analyses to elucidate clinicopathological parameters that predict the patients who will achieve a last PSA value of <0.2.ng/mL and a normal testosterone level at the last follow-up.

We also evaluated the biochemical recurrence (BCR)-free rate using both the Phoenix definition and the definition of PSA ≥ 0.2 ng/mL (the same definition as for radical prostatectomy). If the PSA value after treatment reached 0.2 ng/mL or lower and showed a confirmatory PSA of 0.2 ng/mL or higher, the patient was defined as having BCR the first time an increase in PSA was noted. If the PSA value did not fall to below 0.2 ng/mL, the patient was defined as having BCR at the time of treatment.

A pathologist (N.K.), an expert in prostate cancer diagnosis, centrally reviewed the Gleason score of all biopsy specimens. This study was performed in compliance with the Helsinki Declaration. The Medical Ethics Committee of Nara Medical University approved this retrospective study, and it was exempted from obtaining informed consent from the patients in consideration of the aim and methods of the study.

### Treatment

Among the 445 patients who underwent LDR-BT, 267 patients received neither neoadjuvant nor adjuvant ADT, 141 received neoadjuvant ADT, 10 received adjuvant ADT, and 27 received both neoadjuvant and adjuvant ADT. The radiation consisted of only I-125 seed implantation in 300 patients, and combination treatment including external beam radiation therapy (EBRT) in 145 patients. During the study period, we used three-dimensional conformal radiation therapy for combination therapy. On the other hand, in the IMRT group, 34 patients received neither neoadjuvant nor adjuvant ADT, 24 received neoadjuvant ADT, 14 received adjuvant ADT, and 93 received both neoadjuvant and adjuvant ADT (Table 1). Neoadjuvant ADT was continued for 4 months and adjuvant ADT for 2 years, both in the LDR-BT and IMRT groups. In the IMRT group, concomitant (8-week) ADT was also continued during the radiation period.

Risk was classified according to the modified D’Amico’s risk classification [10]. Patients with clinical stage T3 were classified as “high risk.” The numbers of low-, intermediate-, and high-risk patients were 184, 201, and 60 in the LDR-BT group, and 17, 36, and 112 patients in the IMRT group, respectively.

The prescribed dose of LDR-BT and IMRT are shown in Table 1. In the IMRT group, the prescribed dose was 74 Gy / 37 fractions to 76 Gy/ 38 fractions. All patients were treated by dynamic arc therapy with a micromultileaf collimator (Novalis, BrainLAB A.G., Heimstetten, Germany), and image-guided radiation therapy using infrared-reflecting skin marker positioning and stereoscopic X-ray imaging was adopted (ExacTrac rsp. Novalis Body, BrainLAB A.G., Heimstetten, Germany). In the LDR-BT group, low-risk patients (cT2a, Gleason score 6, and PSA ≤ 10 ng/mL) and intermediate-risk patients (cT2a and PSA ≤ 10 ng/mL) with a Gleason score of 3 + 4 and a positive biopsy core of less than 50% were treated by seed implantation alone and the prescribed dose was 145 Gy or 160 Gy (since November 2008). The other patients received combination treatment including EBRT. The prescribed dose was 110 Gy. The target for EBRT was determined 1 month after seed implantation, and the patients received 45 Gy (in 25 fractions of 1.8 Gy per fraction) using 10 MV photon energy. The clinical target volume included both the whole prostate and one third of the proximal seminal vesicle. Among all patients, a preplanning method was used in 66 patients, an intraoperative planning method in 149 patients, and a real-time planning method in 230 patients. Seed implantation was performed by modified peripheral loading or peripheral loading techniques using Mick’s applicator [11].

### Post-implant dosimetric evaluation

The therapeutic planning and post-implant dosimetric evaluation were performed by one radiation oncologist (I.A.) at 1 month after seed implantation. The dosimetric parameters included the values of the minimal percentage of the dose received by 90% of the prostate gland (%D90), the percentage prostate volume receiving 100% of the prescribed minimal peripheral dose (V100), the minimal percentage of the dose received by 30% of the urethra (%UD30), and the rectal volume receiving 100% of the prescribed dose (R100). The biologically effective dose (BED) was calculated to evaluate an independent factor that can predict BCR, using an α/β ratio of 2 [12]. Implant dose was defined as D90 (dose delivered to 90% of the gland) based on dose-volume histograms. A linear-quadratic model was used to determine BED. The BED values of LDR-BT in combination with EBRT were calculated by adding the BED of both LDR-BT and EBRT [12].

### Statistical analysis

The statistical difference in PSA value at the last follow-up between the LDR-BT group and the IMRT group for categorical variables was tested by the chi-square test, while that for continuous variables was tested by the Mann-Whitney U test and the t-test. The BCR-free rate was estimated by the Kaplan-Meier method. The log-rank test was used for between-group comparison. Univariate and multivariate analyses were conducted by logistic regression analysis. All statistical analyses were performed using PASW Statistics 17.0 (SPSS Inc., Chicago, IL, USA). All *p* values of less than 0.05 were considered statistically significant.

## Results

The distribution of the PSA value at the last follow-up in both the LDR-BT and IMRT groups is shown in Table 2. The achievement rate of PSA < 0.2 ng/mL at the last follow-up was 77.5% in the LDR-BT group and 49.7% in the IMRT group. The LDR-BT group showed significantly lower PSA values at the last follow-up than the IMRT group (*p* < 0.001).

**Table 2:** Number of patients and proportion stratified by PSA value at the last follow-up

	LDR-BT (*n* = 445)		IMRT (*n* = 165)	
PSA (ng/mL)	n	%	n	%
< 0.2	345	77.5	82	49.7
0.2–0.49	39	8.8	35	21.2
0.5–0.99	17	3.8	15	9.1
1.0-	16	3.6	8	4.8
Nadir + 2	28	6.3	25	5.2

*p*< 0.001

To exclude the effect of the testosterone level on PSA fluctuations, we evaluated the PSA value at the last follow-up in patients who showed a normal testosterone level at the last follow-up (Table 3). Three hundred eighty-nine patients (87.4%) in the LDR-BT group and 84 patients (50.9%) in the IMRT group showed a normal testosterone level at the fast follow-up. The achievement rate of PSA < 0.2 ng/mL at the last follow-up was 79.2% in the LDR-BT group and 32.1% in the IMRT group. The LDR-BT group showed a significantly lower PSA value at the last follow-up than the IMRT group (*p* < 0.001) in patients who showed a normal testosterone level at the last follow-up.

**Table 3:** Number of patients with normal testosterone and proportion stratified by PSA value at the last follow-up

	LDR-BT (*n* = 389)		IMRT (*n* = 84)	
PSA (ng/mL)	n	%	n	%
< 0.2	308	79.2	27	32.1
0.2–0.49	39	10.0	28	33.3
0.5–0.99	17	4.4	11	13.1
1.0-	14	3.6	7	8.3
Nadir + 2	11	2.8	11	13.1

*p*< 0.001

We also evaluated the PSA value at the last follow-up after at least 4 years. The difference between the LDR-BT and IMRT groups was significant. The achievement rate of PSA < 0.2 ng/mL at the last follow-up was 79.4% in the LDR-BT group and 45.7% in the IMRT group (Table 4), respectively. The achievement rate of PSA < 0.2 ng/mL in patients who showed a normal testosterone level at the last follow-up was 81.4% in the LDR-BT group and 30.8% in the IMRT group, respectively (Table 5).

**Table 4:** Number of patients and proportion stratified by PSA value at the last follow-up (after at least 4 years)

	LDR-BT (*n* = 412)		IMRT (*n* = 140)	
PSA (ng/mL)	n	%	n	%
< 0.2	327	79.4	64	45.7
0.2–0.49	34	8.3	33	23.6
0.5–0.99	13	3.2	14	10.0
1.0-	10	2.4	7	5.0
Nadir + 2	28	6.8	22	15.7

*p*< 0.001

**Table 5:** Number of patients with normal testosterone and proportion stratified by PSA value at the last follow-up (after at least 4 years)

	LDR-BT (*n* = 360)		IMRT (*n* = 78)	
PSA (ng/mL)	n	%	n	%
< 0.2	293	81.4	24	30.8
0.2–0.49	34	9.4	27	34.6
0.5–0.99	13	3.6	10	12.8
1.0-	9	2.5	6	7.7
Nadir + 2	11	3.1	11	14.1

*p*< 0.001

The 5- and 10-year overall survival rates in the LDR-BT group were 95.3 and 95.1%, and those in the IMRT group were 92.8 and 86.9%, respectively. There was not a significant difference between the two groups (*p* = 0.225). The 5- and 10-year cancer-specific survival rates in the LDR-BT group were 99.8 and 98.9%, and those in the IMRT group were 99.2 and 99.2%, respectively. There was not a significant difference between the two groups (*p* = 0.672). The 5- and 10-year clinical recurrence-free survival rates in the LDR-BT group were 97.8 and 96.0%, and those in the IMRT group were 95.8 and 93.8%, respectively. There was not a significant difference between the two groups (*p* = 0.164).

**Fig. 1:**
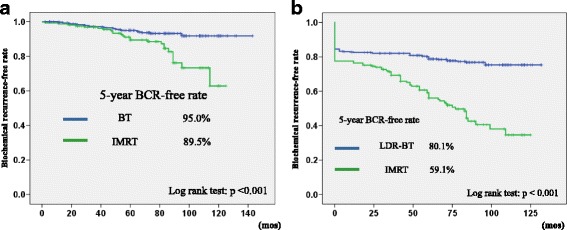
**a** Biochemical recurrence-free rate using the Phoenix definition **b** Biochemical recurrence-free rate using the definition of PSA ≥ 0.2 ng/mL

Using a Phoenix definition, the 5-year BCR-free rate in the LDR-BT and IMRT groups was 95.0 and 89.5% (*p* < 0.001), respectively (Fig. 1a). On the other hand, the 5-year BCR-free rate in the LDR-BT and IMRT groups using the definition of PSA ≥ 0.2 ng/mL were 80.1 and 59.1% (*p* < 0.001), respectively (Fig. 1b).

**Fig. 2:**
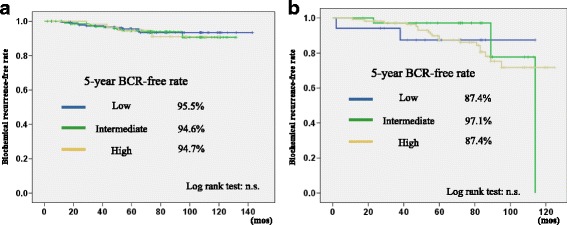
**a** Biochemical recurrence-free rate of the LDR-BT group stratified by D’Amico risk classification using the Phoenix definition. **b** Biochemical recurrence-free rate of the IMRT group stratified by D’Amico risk classification using the Phoenix definition

Regarding risk stratification using the Phoenix definition, the 5-year BCR-free rate in the low, intermediate, and high-risk patients in the LDR-BT group were 95.5, 94.6, and 94.7% (Fig. 2a), respectively, while those in the IMRT group were 87.4, 97.1, and 87.4% (Fig. 2b), respectively. There was not a significant difference in the BCR-free rate between the different risk groups.

**Fig. 3:**
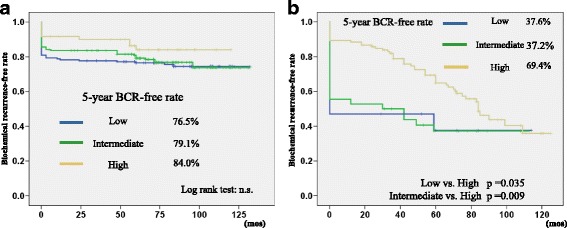
**a** Biochemical recurrence-free rate of the LDR-BT group stratified by D’Amico risk classification using the definition of PSA ≥ 0.2 ng/mL. **b** Biochemical recurrence-free rate of the IMRT group stratified by D’Amico risk classification using the definition of PSA ≥ 0.2 ng/mL

On the other hand, using the definition of PSA ≥ 0.2 ng/mL, the 5-year BCR-free rate in the low, intermediate, and high-risk patients was 76.5, 79.1, and 84.0%, respectively (Fig. 3a), in the LDR-BT group, and 37.6, 37.2, and 69.4%, respectively (Fig. 3b), in the IMRT group. The 5-year BCR-free rate in high-risk patients was significantly higher than that in low- and intermediate-risk patients (high vs. low: *p* = 0.035, and high vs. intermediate: *p* = 0.009). The 5-year BCR-free rate in the low (76.5% vs. 37.6%, *p* = 0.001) intermediate (79.1% vs. 37.2%, *p* < 0.001), and high risk (84.0% vs. 69.4, *p* < 0.001) patients in the LDR-BT group was significantly higher than those in the IMRT group.

Regarding the post-implant dosimetric evaluation of the LDR-BT group, the median (range) value of %D90, V100, %UD30, and R100 were 114.1%(79.8–144.5%), 96.1% (77.8–100%), 136.1% (96.1–200.3%), and 0.02 mL (0.00–2.42 mL), respectively.

**Fig. 4:**
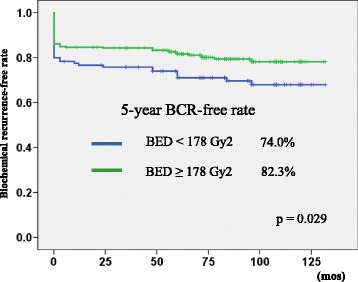
Biochemical recurrence-free rate of the LDR-BT group stratified by biologically effective dose (BED) using the definition of PSA ≥ 0.2 ng/mL

In subgroup analysis, we divided patients by BED to evaluate BCR-free rate (the definition of PSA ≥ 0.2 ng/mL) in the LDR-BT group (Fig. 4). To set the cut-off points of BED, we used receiver-operating characteristic curve analysis. Patients with a higher BED (≥ 178 Gy2) had a significantly higher BCR-free rate than those with a lower BED (< 178 Gy2) (5-year BCR-free rate: 82.3% vs. 74.0%, *p* = 0.029).

Table 6 shows the results of univariate and multivariate logistic regression analyses predicting a last PSA value of <0.2 ng/mL with a normal testosterone level at the last follow-up (after at least 4 years). In univariate analysis, treatment modality (IMRT vs. LDR-BT), PSA (≤ 10 ng/mL vs. > 20 ng/mL), BED (< 160 Gy2 vs. One hundred sixty to One hundred eighty Gy2, and 180 Gy2 ≤), age (≤ 64 years vs. Sixty four to seventy five years), risk (low vs. high), and ADT use (none vs. neoad+/ad+) were significant parameters predicting a last PSA value of <0.2 ng/mL with a normal testosterone level at the last follow-up (after at least 4 years). In the multivariate analysis, LDR-BT, higher BED and older patients remained independent parameters.

**Table 6:** Logistic regression analysis predicting a last PSA value of <0.2 ng/mL in patients with a normal testosterone level at the last follow-up (after at least 4 years)

		univariate			multivariate		
variables		OR	95%C.I.	*P*-value	OR	95%C.I.	*P*-value
IMRT (reference) vs. BT		9.840	5.681–17.041	<0.001	5.032	1.911–13.253	0.001
PSA	-10 ng/mL	reference					n.s.
	10-20 ng/mL	0.766	0.459–1.277	0.306			
	20 ng/mL-	0.202	0.096–0.426	<0.001			
BED	-160Gy2	reference			reference		
	160-180Gy2	7.040	3.546–13.977	<0.001	2.753	1.017–7.450	0.046
	180Gy2-	8.038	4.800–13.459	<0.001	2.859	1.184–6.903	0.020
Age	−64	reference			reference		
	65–74	1.666	1.017–2.730	0.043	2.679	1.524–4.709	0.001
	75-	1.498	0.819–2.742	0.190	2.956	1.444–6.053	0.003
Risk (D’Amico)	Low	reference					n.s.
	Intermediate	0.982	0.604–1.597	0.941			
	High	0.445	0.255–0.778	0.004			
ADT use	none	reference					n.s.
	Neoad+	0.729	0.450–1.183	0.201			
	Ad+	0.290	0.070–1.197	0.087			
	Neoad+/Ad+	0.326	0.175–0.610	<0.001			

*OR* odds ratio, *C.I* confidential interval, *IMRT* intensity modulated radiation therapy, *BT* brachytherapy, *PSA* prostate specific antigen, *BED* biological effective dose, *ADT* androgen deprivation therapy, *Neoad* neoadjuvant, *Ad* adjuvant

## Discussion

LDR-BT has come to be widely used as a definitive treatment modality for prostate cancer, not only for low-risk patients, but also for intermediate and high-risk patients in recent years. At present, the oncologic outcome of patients who undergo LDR-BT is reportedly similar to that of patients who undergo IMRT as well as radical prostatectomy [1–6]. However, the definition of recurrence is different for prostatectomy and for radiation therapy. It is inappropriate to compare the oncologic outcome using different definitions. To address this issue, Critz et al. reported the long-term oncologic outcomes in patients who underwent LDR-BT in combination with EBRT using the same definition of surgery (the definition of PSA ≥ 0.2 ng/mL) [8]. This is the first report to compare LDR-BT with radical prostatectomy using the same definition. The long-term oncologic outcomes were similar between LDR-BT and surgery. We also reported the oncologic outcome in patients who underwent LDR-BT using the definition of PSA ≥ 0.2 ng/mL [9]. Approximately 80% of patients showed a low PSA value of below 0.2 ng/mL at the last follow-up.

In the present study, we demonstrated that the PSA value at the follow-up is significantly different for LDR-BT and IMRT. Indeed, 77.5% of the LDR-BT group showed PSA < 0.2 ng/mL at the last follow-up, while only 49.7% of the IMRT group did (Table 2). However, neoadjuvant ADT and/or adjuvant ADT (2–3 years) is often used in patients who undergo LDR-BT or IMRT for the oncologic effect and /or size reduction of the prostate. Long-term ADT affects recovery of the testosterone level. We also elucidated the influence of the testosterone level in this study (Table 3). After eliminating the effect of ADT by focusing on patients with a normal level of testosterone, 79.2% of patients in the LDR-BT group achieved PSA < 0.2 ng/mL, compared to only 32.1% in the IMRT group (*p* < 0.001). We also focused on the PSA value at the last follow-up of patients with a follow-up period of at least 4 years. This trend was significant (Tables 4, 5).

Jabbari et al. reported a significant difference in the nadir PSA value between LDR-BT and three-dimensional conformal radiation therapy, and also between LDR-BT and conformal proton beam radiotherapy [13]. Previous reports suggested that achievement of a lower PSA value after LDR-BT promises a more favorable oncologic outcome [14, 15]. Ko et al. showed that patients with a PSA nadir of <0.5 ng/mL after LDR-BT had significantly higher long-term freedom from biochemical failure and higher freedom from distant metastases [14]. Lo et al. illustrated that patients with 48-month PSA ≤ 0.4 ng/mL had a < 1% risk of disease relapse at 8 years after LDR-BT, whereas all patients with 48-month PSA > 1.0 ng/mL relapsed [15]. Stone et al. reported that patients with higher BED showed significant lower BCR [16]. BED is also the only predictive parameter of cancer-specific survival in multivariate analysis [16]. The BED of LDR-BT, especially in case of combination with EBRT, is significantly higher than that of IMRT. Indeed, Zelefsky et al. reported that the BCR-free rate using the Phoenix definition was significantly lower in patients who underwent IMRT (81 Gy) than in patients who underwent LDR-BT among patients with favorable risk prostate cancer [17]. They also showed the same result for intermediate risk patients who received ultra-high dose IMRT (86.4 Gy) compared with patients who received combined brachytherapy and IMRT [18]. The recently published results of the ASCENDE-RT trial also supported the advantage of LDR-BT boost compared to dose-escalated EBRT (78Gy) in respect to biochemical failure [19]. Previous reports mentioned above and our present study support the fact that patients with a higher BED can achieve a lower PSA nadir, and expect a lower BCR.

In the present study, we demonstrated that most patients (86.3%) who underwent LDR-BT achieved PSA < 0.5 ng/mL with a median follow-up period of 75 months. Patients who achieved a higher BED of ≥178 Gy2 also showed a favorable BCR-free rate (82.3%) using the definition of PSA ≥ 0.2 ng/mL in LDR-BT patients (Fig. 4).

The univariate logistic analysis showed that treatment modality (IMRT vs. LDR-BT), initial PSA, BED, age, risk classification and ADT use were significant parameters that predicted a last PSA value of <0.2 ng/mL in patients with a normal testosterone level at the last follow-up after at least 4 years (Table 6). In the multivariate analysis, LDR-BT, higher BED, and older patients remained independent parameters to achieve PSA value <0.2 ng/mL. The reason why older patients can achieve a lower PSA value at the last follow-up is uncertain. Potentially, several factors such as sexual activity, PSA bounce, and radiation sensitivity are conceivable. On the other hand, it is reasonable that a higher local radiation dose (BED) can be obtained with LDR-BT than IMRT, playing an important role to achieve a lower PSA value at the last follow-up.

There are several limitations to this study. Firstly, the number of patients is small. Secondly, the follow-up period is short. Indeed, some patients showed BCR (7%) at 10 years after treatment, as reported by Critz et al. [8]. Thirdly, patient characteristics were different between the two groups. For example, half of the IMRT group showed a lower than normal testosterone level at the last follow-up, while 87% of the LDR-BT group showed a normal testosterone level. Fourthly, we used infrared-reflecting skin marker (not fiducial markers inserted into prostate) as IGRT for IMRT. Fifthly, this study is not a randomized controlled trial. Direct comparison of BCR-free rate is not appropriate. Under these limitations, we set the main purpose of this study comparison of the PSA value at the last follow-up. However, the PSA value of the LDR-BT group at the last follow-up is significantly lower. Further evaluation with a longer follow-up period has to demonstrate the advantage of LDR-BT on oncologic outcome.

## Conclusions

The PSA value at the last follow-up of LDR-BT was significantly lower than that of IMRT, and this result was particularly distinct in patients with a normal testosterone level at the last follow-up.

## Abbreviations

%D90: Minimal percentage of the dose received by 90% of the prostate gland
%UD30: Minimal percentage of the dose received by 30% of the urethra
ADT: Androgen deprivation therapy
BCR: Biochemical recurrence
BED: Biologically effective dose
EBRT: External beam radiation therapy
IMRT: Intensity-modulated radiation therapy
LDR-BT: Low-dose rate brachytherapy
PSA: Prostate specific antigen
R100: Rectal volume receiving 100%of the prescribed dose
V100: Percentage prostate volume receiving 100% of the prescribed minimal peripheral dose
